# Supplementary material for: A Complex In Vitro Degradation Study on Polydioxanone Biliary Stents during a Clinically Relevant Period with the Focus on Raman Spectroscopy Validation
Source: Polymers (Basel). 2022 Feb 26;14(5):938. doi: 10.3390/polym14050938 (PMC8912347; doi:10.3390/polym14050938)
Supplement: Supplementary file 1 [file polymers-14-00938-s001.zip › Text S1.pdf]

Calculation procedure of the area under the 1732 cm^{-1} Raman peak shoulder

The area under the shoulder of the 1732 cm^{-1} peak in Raman spectrum was calculated in the following way:

At first, the baseline was removed from each spectrum to allow a relevant quantification of the 1732 cm^{-1} peak height and the adjacent shoulder area. Then, for each of the 32 spectra acquired in the grid shown in Figure 2, a non-normalized shoulder area (between 1736 and 1757 cm^{-1}) was calculated simply as a sum of vertical slices below the curve:

$$\sum_{i=1}^{n} (\Delta v_i \cdot I_i)$$

where Δv_i is the difference between the adjacent wavenumbers (*i.e.*, $v_{i+1} - v_i$) in the spectral data, and I_i is the corresponding intensity value (*i.e.*, the number of photons detected). The index i indexes wavenumbers (and the corresponding intensity values) in the spectral data, so that it covers the spectral region between 1736 and 1757 cm^{-1}. Since Δv_i is expressed in cm^{-1} and I_i is a dimensionless number, the physical unit of the non-normalized area is cm^{-1}.

This non-normalized area was subsequently normalized by dividing it with the height of the neighboring 1732 cm^{-1} peak. (This height corresponds to the intensity of this peak, again expressed as the number of photons detected.) Expressed mathematically:

$$A_{norm} = \frac{A_{nn}}{h_{1732}}$$

where A_{norm} is the normalized area, A_{nn} is the non-normalized area, and h_{1732} is the height of the 1732 cm^{-1} peak in this spectrum. Since A_{nn} is expressed in cm^{-1} and h_{1732} is a dimensionless number (the number of photons), also the physical unit of the normalized area is cm^{-1}.

The areas calculated in this way were finally averaged, thus obtaining the final value of "normalized peak shoulder area" for the given degradation period.
