# Supplementary material for: A Complex In Vitro Degradation Study on Polydioxanone Biliary Stents during a Clinically Relevant Period with the Focus on Raman Spectroscopy Validation
Source: Polymers (Basel). 2022 Feb 26;14(5):938. doi: 10.3390/polym14050938 (PMC8912347; doi:10.3390/polym14050938)
Supplement: Supplementary file 1 [file polymers-14-00938-s001.zip › Dataset-S1.zip!/stenty_DSC/stenty_DSC_0/ZZ - UHK-0-1-2.001.pdf]

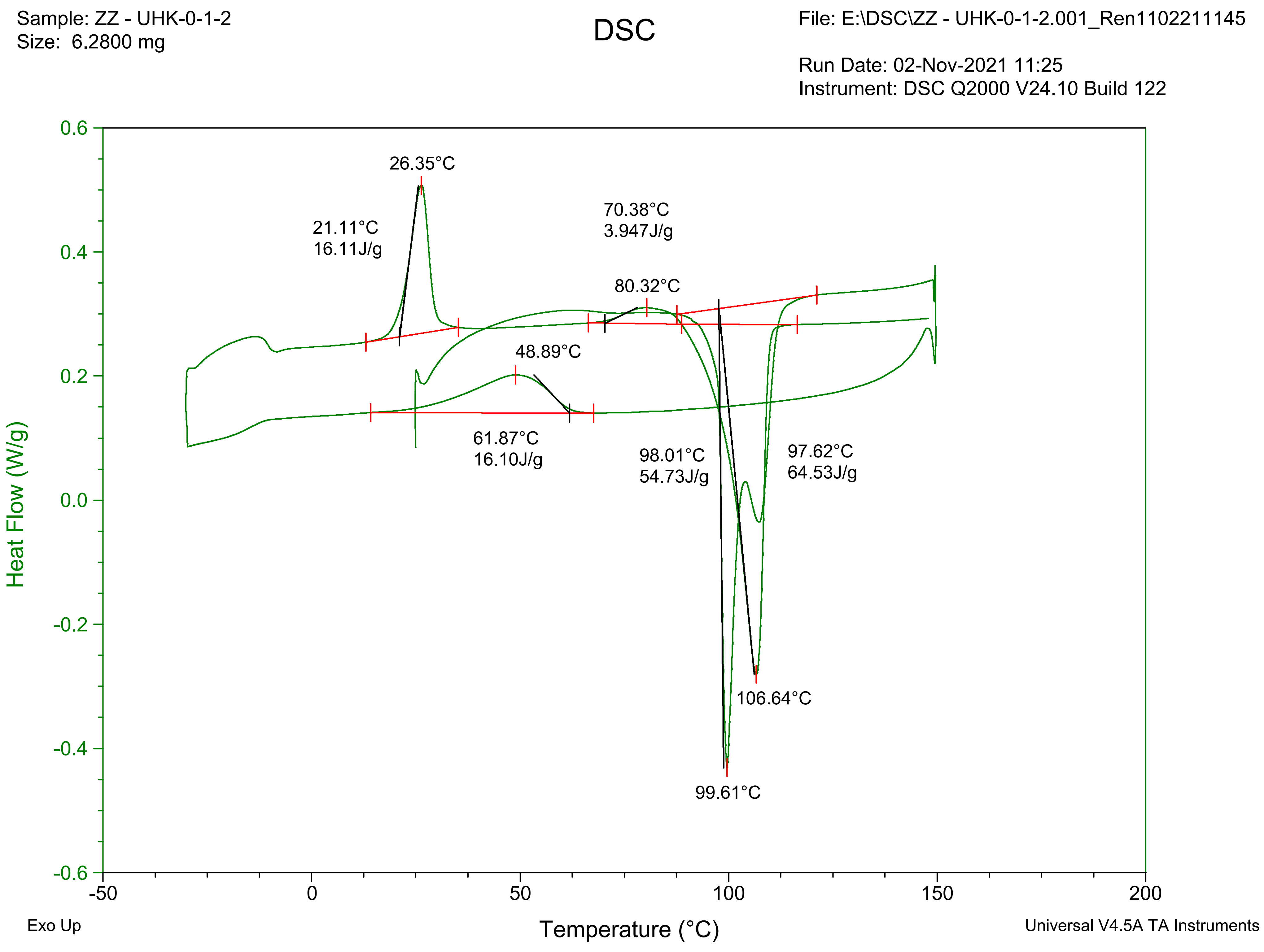

Sample: ZZ - UHK-0-1-2
Size: 6.2800 mg
DSC
File: E:\DSC\ZZ - UHK-0-1-2.001_Ren1102211145
Run Date: 02-Nov-2021 11:25
Instrument: DSC Q2000 V24.10 Build 122
26.35°C
21.11°C
16.11J/g
70.38°C
3.947J/g
80.32°C
48.89°C
61.87°C
16.10J/g
98.01°C
54.73J/g
97.62°C
64.53J/g
106.64°C
99.61°C
Heat Flow (W/g)
0.6
0.4
0.2
0.0
-0.2
-0.4
-0.6
-50
0
50
100
150
200
Temperature (°C)
Exo Up
Universal V4.5A TA Instruments
