# Supplementary material for: A Complex In Vitro Degradation Study on Polydioxanone Biliary Stents during a Clinically Relevant Period with the Focus on Raman Spectroscopy Validation
Source: Polymers (Basel). 2022 Feb 26;14(5):938. doi: 10.3390/polym14050938 (PMC8912347; doi:10.3390/polym14050938)
Supplement: Supplementary file 1 [file polymers-14-00938-s001.zip › Dataset-S1.zip!/stenty_DSC/stenty_DSC_0/ZZ - UHK-0-5-2.001.pdf]

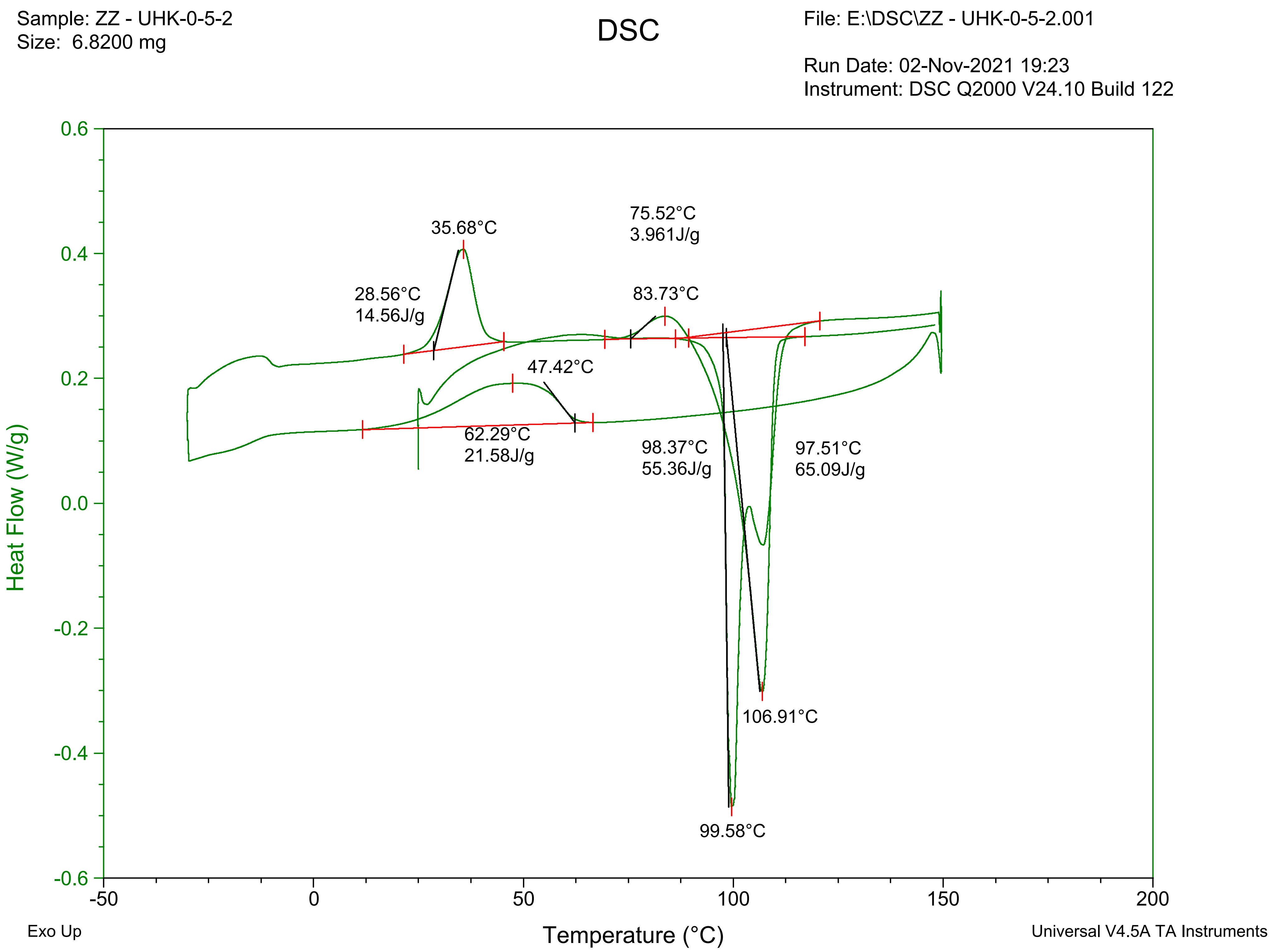
Sample: ZZ - UHK-0-5-2
Size: 6.8200 mg
DSC
File: E:\DSC\ZZ - UHK-0-5-2.001
Run Date: 02-Nov-2021 19:23
Instrument: DSC Q2000 V24.10 Build 122
35.68°C
75.52°C
3.961J/g
28.56°C
14.56J/g
83.73°C
47.42°C
62.29°C
21.58J/g
98.37°C
55.36J/g
97.51°C
65.09J/g
106.91°C
99.58°C
0.6
0.4
0.2
0.0
-0.2
-0.4
-0.6
Heat Flow (W/g)
-50
0
50
100
150
200
Temperature (°C)
Exo Up
Universal V4.5A TA Instruments
