# Supplementary material for: A Complex In Vitro Degradation Study on Polydioxanone Biliary Stents during a Clinically Relevant Period with the Focus on Raman Spectroscopy Validation
Source: Polymers (Basel). 2022 Feb 26;14(5):938. doi: 10.3390/polym14050938 (PMC8912347; doi:10.3390/polym14050938)
Supplement: Supplementary file 1 [file polymers-14-00938-s001.zip › Dataset-S1.zip!/stenty_DSC/stenty_DSC_3t/ZZ - UHK-3-2-2.001.pdf]

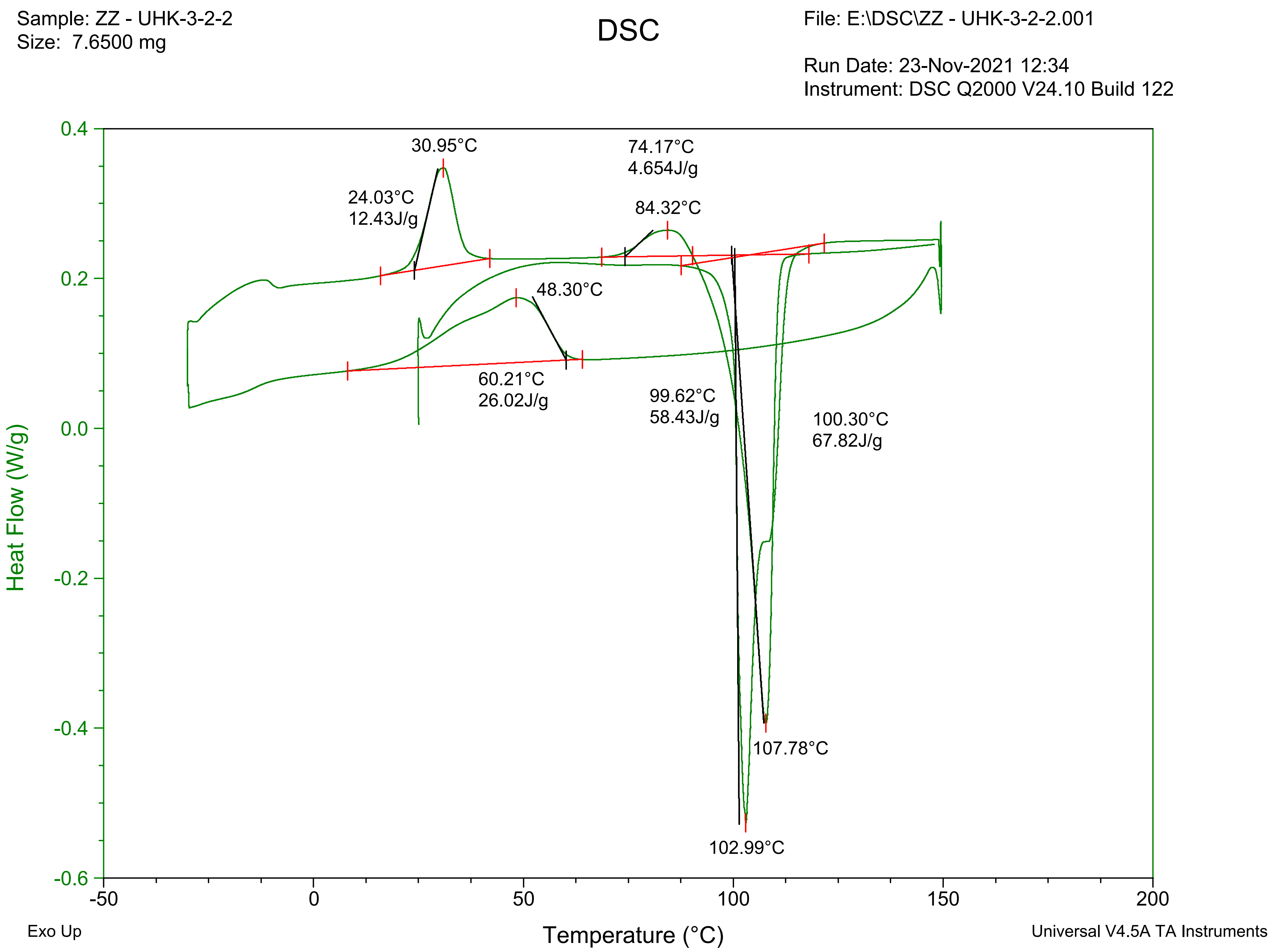

Sample: ZZ - UHK-3-2-2
Size: 7.6500 mg
DSC
File: E:\DSC\ZZ - UHK-3-2-2.001
Run Date: 23-Nov-2021 12:34
Instrument: DSC Q2000 V24.10 Build 122
30.95°C
24.03°C
12.43J/g
74.17°C
4.654J/g
84.32°C
48.30°C
60.21°C
26.02J/g
99.62°C
58.43J/g
100.30°C
67.82J/g
107.78°C
102.99°C
Heat Flow (W/g)
0.4
0.2
0.0
-0.2
-0.4
-0.6
-50
0
50
100
150
200
Temperature (°C)
Exo Up
Universal V4.5A TA Instruments
