# Supplementary material for: A Complex In Vitro Degradation Study on Polydioxanone Biliary Stents during a Clinically Relevant Period with the Focus on Raman Spectroscopy Validation
Source: Polymers (Basel). 2022 Feb 26;14(5):938. doi: 10.3390/polym14050938 (PMC8912347; doi:10.3390/polym14050938)
Supplement: Supplementary file 1 [file polymers-14-00938-s001.zip › Dataset-S1.zip!/stenty_DSC/stenty_DSC_3t/ZZ - UHK-3-5-2.001.pdf]

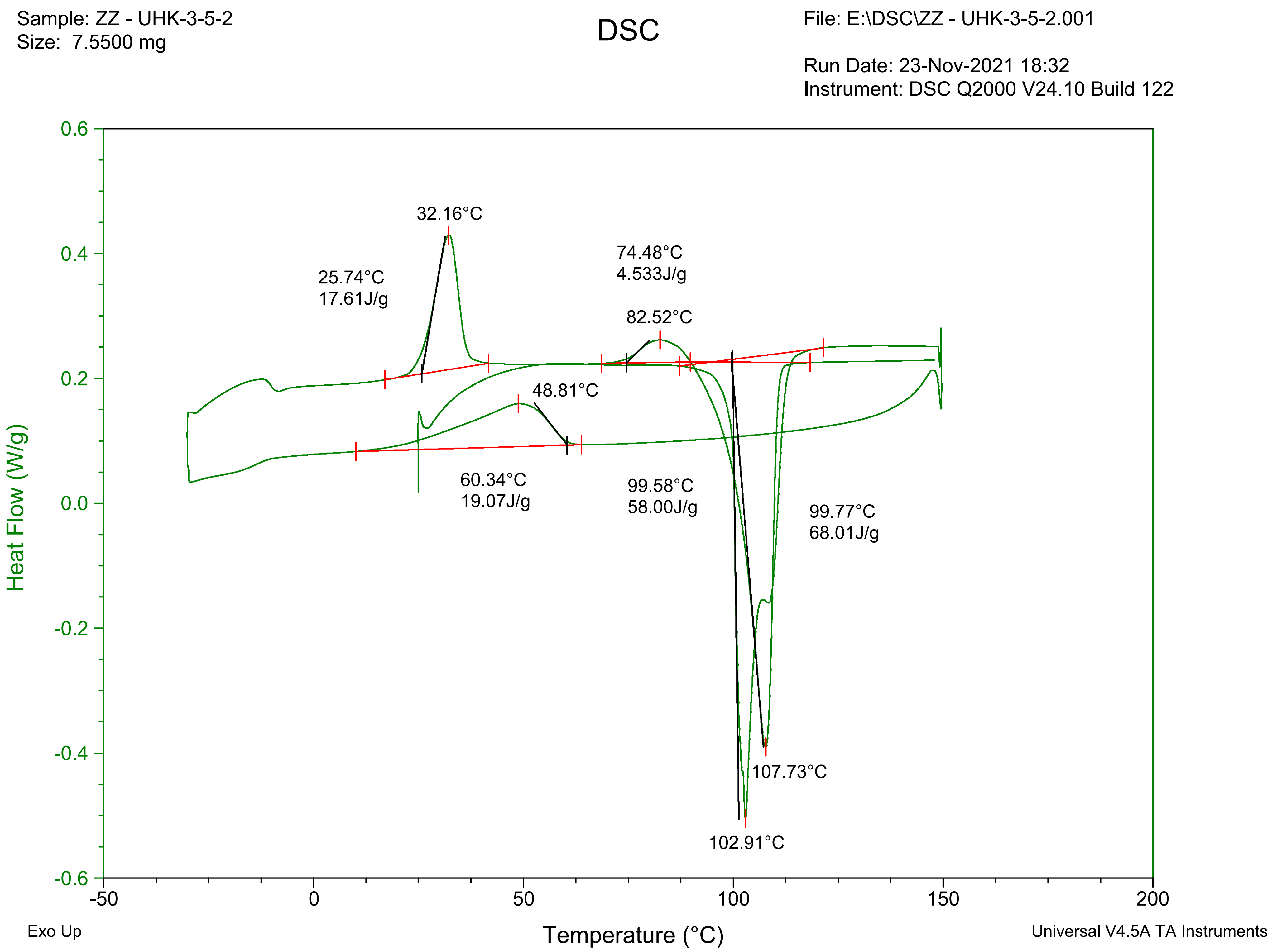

Sample: ZZ - UHK-3-5-2
Size: 7.5500 mg
DSC
File: E:\DSC\ZZ - UHK-3-5-2.001
Run Date: 23-Nov-2021 18:32
Instrument: DSC Q2000 V24.10 Build 122
32.16°C
25.74°C
17.61J/g
74.48°C
4.533J/g
82.52°C
48.81°C
60.34°C
19.07J/g
99.58°C
58.00J/g
99.77°C
68.01J/g
107.73°C
102.91°C
Heat Flow (W/g)
0.6
0.4
0.2
0.0
-0.2
-0.4
-0.6
-50
0
50
100
150
200
Exo Up
Temperature (°C)
Universal V4.5A TA Instruments
