# Supplementary material for: A Complex In Vitro Degradation Study on Polydioxanone Biliary Stents during a Clinically Relevant Period with the Focus on Raman Spectroscopy Validation
Source: Polymers (Basel). 2022 Feb 26;14(5):938. doi: 10.3390/polym14050938 (PMC8912347; doi:10.3390/polym14050938)
Supplement: Supplementary file 1 [file polymers-14-00938-s001.zip › Dataset-S1.zip!/stenty_DSC/stenty_DSC_6t/ZZ - UHK-6-1-2.001.pdf]

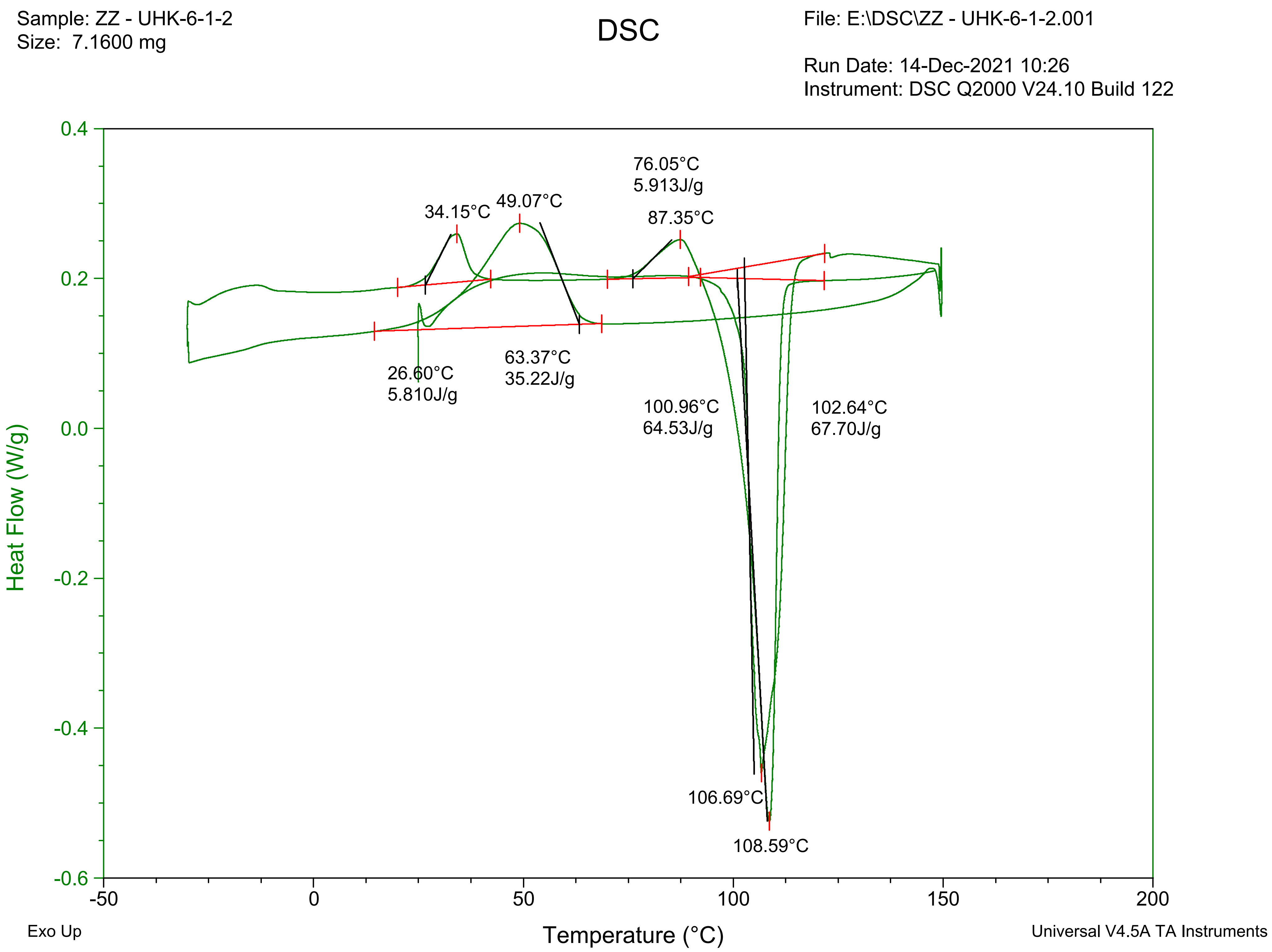

Sample: ZZ - UHK-6-1-2
Size: 7.1600 mg
DSC
File: E:\DSC\ZZ - UHK-6-1-2.001
Run Date: 14-Dec-2021 10:26
Instrument: DSC Q2000 V24.10 Build 122
76.05°C
5.913J/g
34.15°C
49.07°C
87.35°C
26.60°C
5.810J/g
63.37°C
35.22J/g
100.96°C
64.53J/g
102.64°C
67.70J/g
106.69°C
108.59°C
Heat Flow (W/g)
0.4
0.2
0.0
-0.2
-0.4
-0.6
-50
0
50
100
150
200
Temperature (°C)
Exo Up
Universal V4.5A TA Instruments
