# Supplementary material for: A Complex In Vitro Degradation Study on Polydioxanone Biliary Stents during a Clinically Relevant Period with the Focus on Raman Spectroscopy Validation
Source: Polymers (Basel). 2022 Feb 26;14(5):938. doi: 10.3390/polym14050938 (PMC8912347; doi:10.3390/polym14050938)
Supplement: Supplementary file 1 [file polymers-14-00938-s001.zip › Dataset-S1.zip!/stenty_DSC/stenty_DSC_9t/ZZ - UHK-9-4-2.001.pdf]

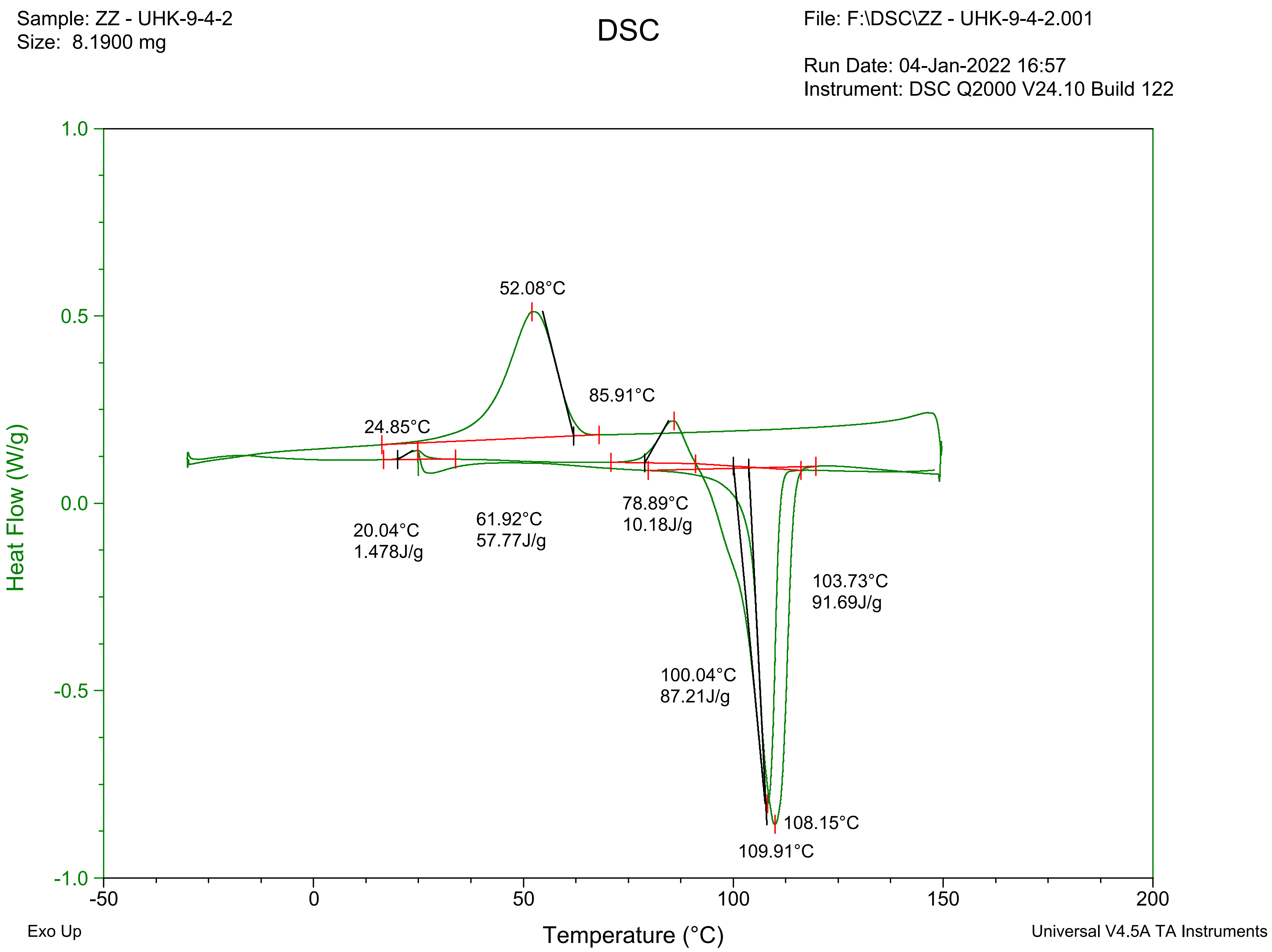
Sample: ZZ - UHK-9-4-2
Size: 8.1900 mg
DSC
File: F:\DSC\ZZ - UHK-9-4-2.001
Run Date: 04-Jan-2022 16:57
Instrument: DSC Q2000 V24.10 Build 122
52.08°C
85.91°C
24.85°C
20.04°C
1.478J/g
61.92°C
57.77J/g
78.89°C
10.18J/g
103.73°C
91.69J/g
100.04°C
87.21J/g
108.15°C
109.91°C
Heat Flow (W/g)
1.0
0.5
0.0
-0.5
-1.0
-50
0
50
100
150
200
Temperature (°C)
Exo Up
Universal V4.5A TA Instruments
