# Supplementary material for: A Complex In Vitro Degradation Study on Polydioxanone Biliary Stents during a Clinically Relevant Period with the Focus on Raman Spectroscopy Validation
Source: Polymers (Basel). 2022 Feb 26;14(5):938. doi: 10.3390/polym14050938 (PMC8912347; doi:10.3390/polym14050938)
Supplement: Supplementary file 1 [file polymers-14-00938-s001.zip › Dataset-S1.zip!/stenty_DSC/stenty_DSC_9t/ZZ - UHK-9-7-2.001.pdf]

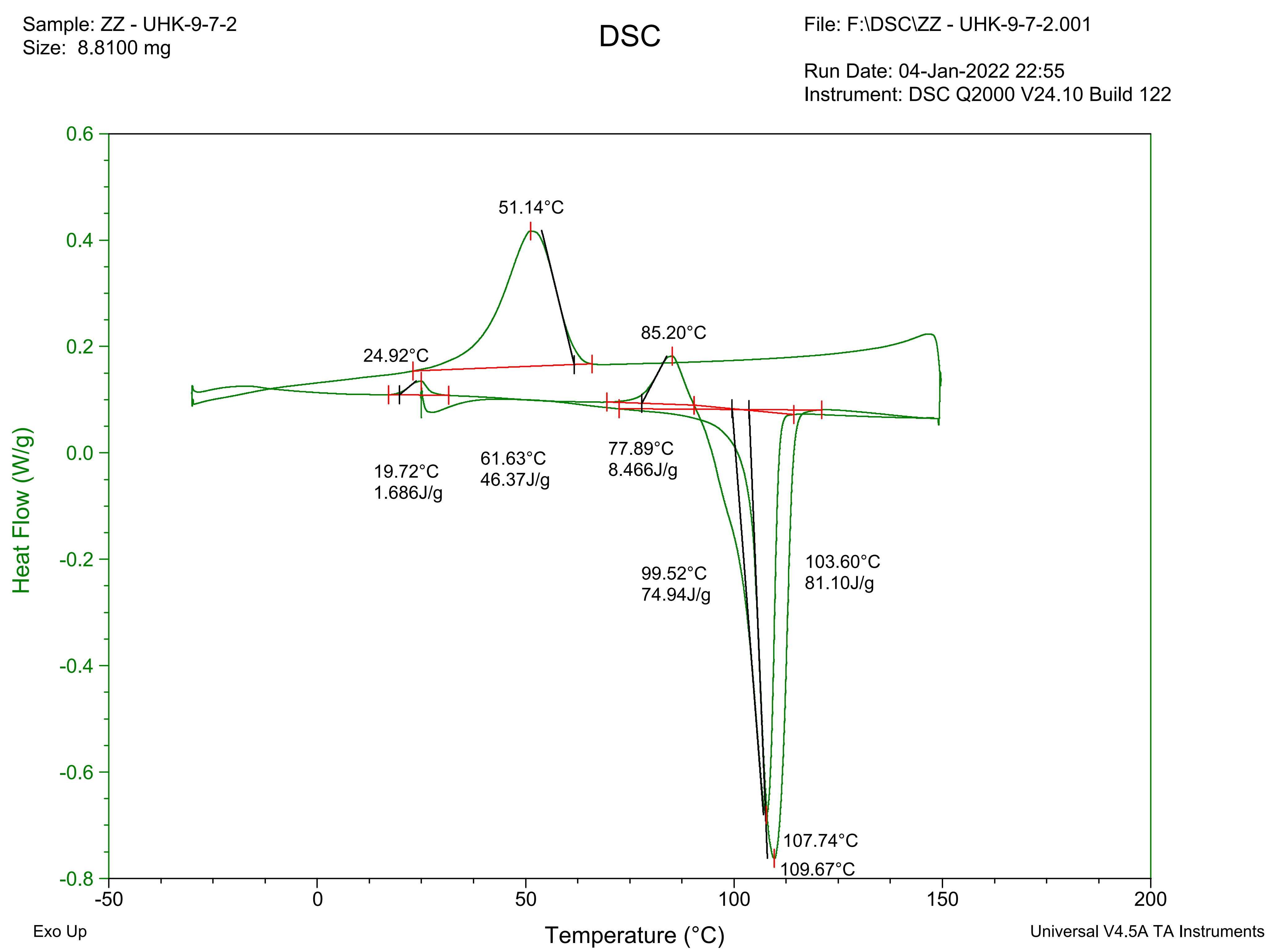
Sample: ZZ - UHK-9-7-2
Size: 8.8100 mg
DSC
File: F:\DSC\ZZ - UHK-9-7-2.001
Run Date: 04-Jan-2022 22:55
Instrument: DSC Q2000 V24.10 Build 122
51.14°C
85.20°C
24.92°C
19.72°C
1.686J/g
61.63°C
46.37J/g
77.89°C
8.466J/g
99.52°C
74.94J/g
103.60°C
81.10J/g
107.74°C
109.67°C
0.6
0.4
0.2
0.0
-0.2
-0.4
-0.6
-0.8
Heat Flow (W/g)
-50
0
50
100
150
200
Temperature (°C)
Exo Up
Universal V4.5A TA Instruments
