# Supplementary material for: A Complex In Vitro Degradation Study on Polydioxanone Biliary Stents during a Clinically Relevant Period with the Focus on Raman Spectroscopy Validation
Source: Polymers (Basel). 2022 Feb 26;14(5):938. doi: 10.3390/polym14050938 (PMC8912347; doi:10.3390/polym14050938)
Supplement: Supplementary file 1 [file polymers-14-00938-s001.zip › Dataset-S1.zip!/stenty_DSC/stenty_DSC_9t/ZZ - UHK-9-8-2.001.pdf]

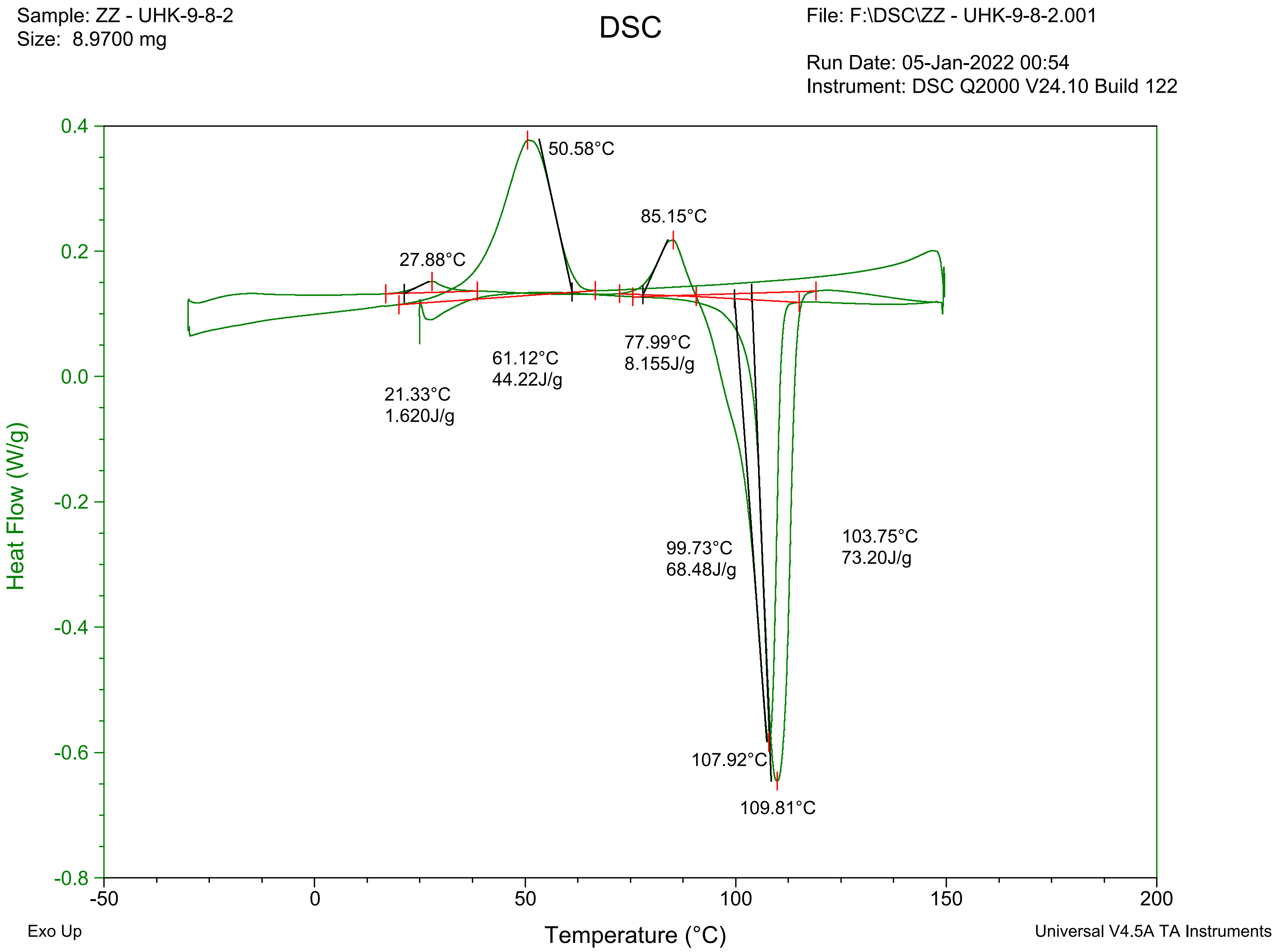
Sample: ZZ - UHK-9-8-2
Size: 8.9700 mg
DSC
File: F:\DSC\ZZ - UHK-9-8-2.001
Run Date: 05-Jan-2022 00:54
Instrument: DSC Q2000 V24.10 Build 122
50.58°C
85.15°C
27.88°C
61.12°C
44.22J/g
77.99°C
8.155J/g
21.33°C
1.620J/g
99.73°C
68.48J/g
103.75°C
73.20J/g
107.92°C
109.81°C
Heat Flow (W/g)
0.4
0.2
0.0
-0.2
-0.4
-0.6
-0.8
-50
0
50
100
150
200
Temperature (°C)
Exo Up
Universal V4.5A TA Instruments
