# Supplementary material for: A Complex In Vitro Degradation Study on Polydioxanone Biliary Stents during a Clinically Relevant Period with the Focus on Raman Spectroscopy Validation
Source: Polymers (Basel). 2022 Feb 26;14(5):938. doi: 10.3390/polym14050938 (PMC8912347; doi:10.3390/polym14050938)
Supplement: Supplementary file 1 [file polymers-14-00938-s001.zip › Dataset-S1.zip!/stenty_DSC/stenty_DSC_Tg/ZZ_-_UHK-9-2-2.001.pdf]

Sample: ZZ - UHK-9-2-2
Size: 8.8600 mg

DSC

File: F:\temp2\ZZ - UHK-9-2-2.001

Run Date: 04-Jan-2022 12:58
Instrument: DSC Q2000 V24.10 Build 122

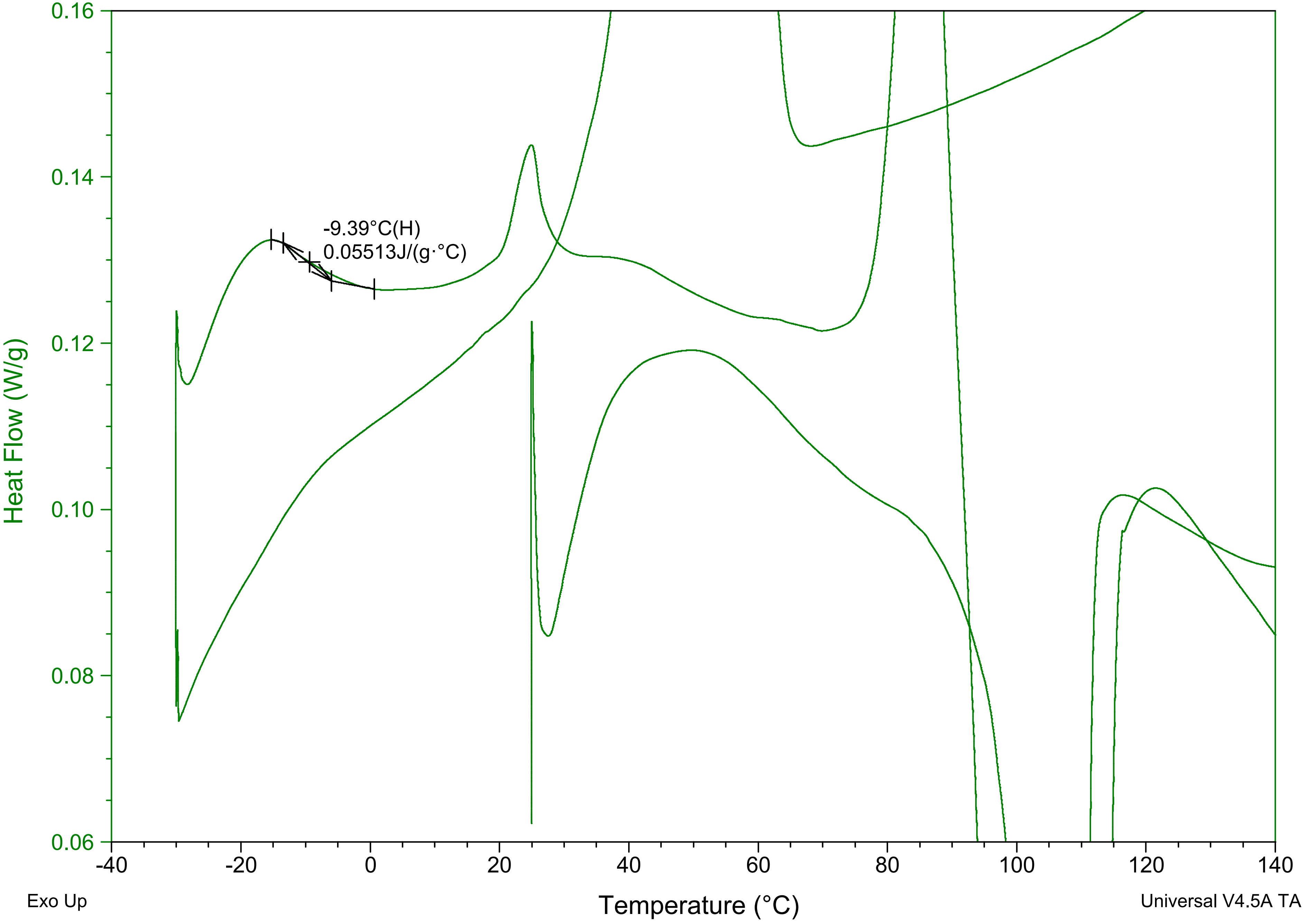

Exo Up

Universal V4.5A TA Instruments
