# Supplementary material for: A Complex In Vitro Degradation Study on Polydioxanone Biliary Stents during a Clinically Relevant Period with the Focus on Raman Spectroscopy Validation
Source: Polymers (Basel). 2022 Feb 26;14(5):938. doi: 10.3390/polym14050938 (PMC8912347; doi:10.3390/polym14050938)
Supplement: Supplementary file 1 [file polymers-14-00938-s001.zip › Dataset-S1.zip!/stenty_DSC/stenty_DSC_Tg/ZZ_-_UHK-9-3-2.001.pdf]

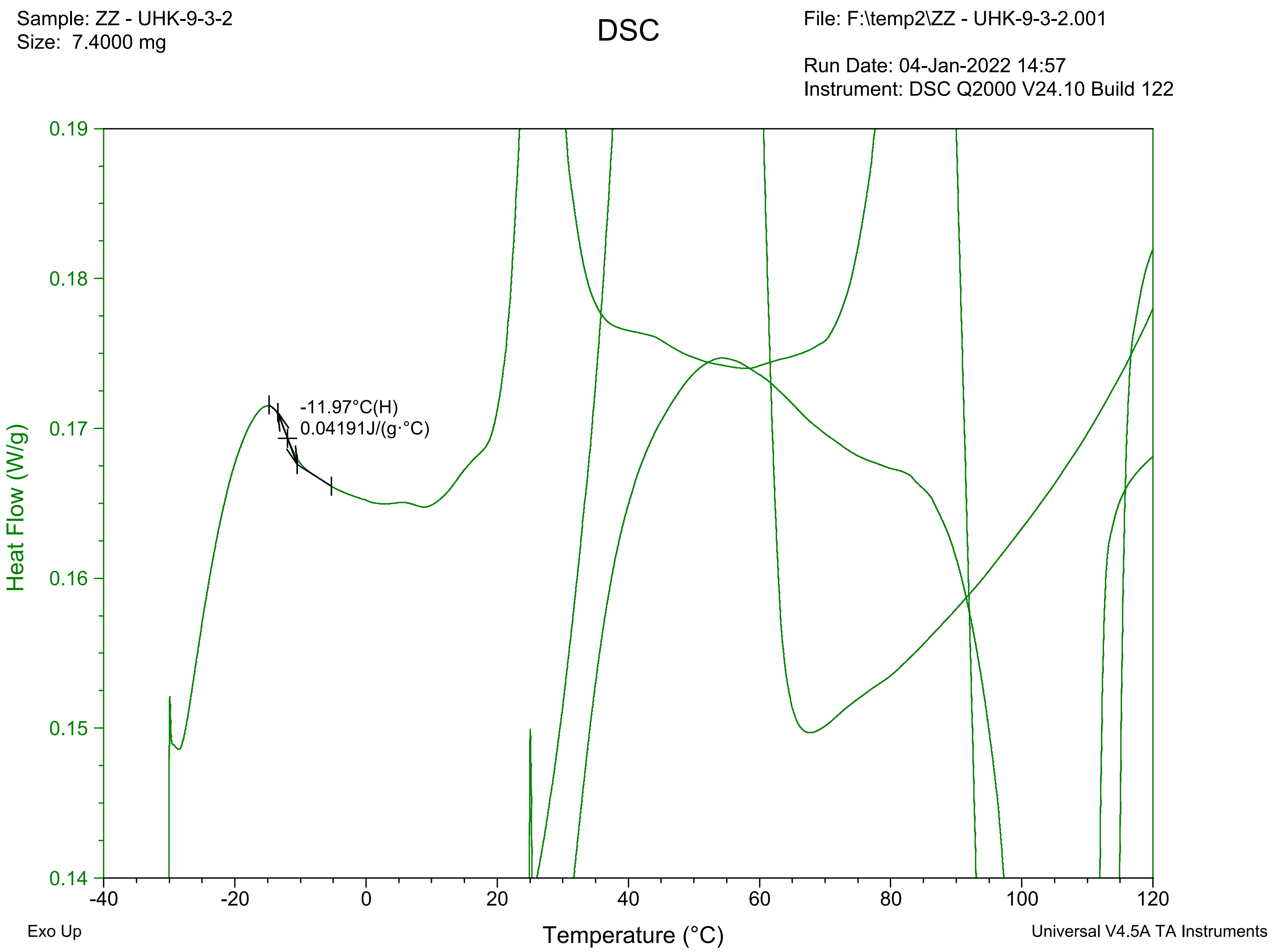
Sample: ZZ - UHK-9-3-2
Size: 7.4000 mg
DSC
File: F:\temp2\ZZ - UHK-9-3-2.001
Run Date: 04-Jan-2022 14:57
Instrument: DSC Q2000 V24.10 Build 122
-11.97°C(H)
0.04191J/(g·°C)
Heat Flow (W/g)
0.19
0.18
0.17
0.16
0.15
0.14
-40
-20
0
20
40
60
80
100
120
Temperature (°C)
Exo Up
Universal V4.5A TA Instruments
